# Assessment of Upper Limb Function Using Virtual Reality Technologies in Adults with Neurological Disorders: A Systematic Review

**DOI:** 10.3390/neurolint18080142

**Published:** 2026-07-24

**Authors:** José Bel-Lacoma, Ángela Aguilera-Rubio, Roberto Cano-de-la-Cuerda

**Affiliations:** 1 International Doctorate School, Rey Juan Carlos University, 28933 Madrid, Spain; j.bel.2024@alumnos.urjc.es; 2 Fundación Tobías, Ronda Hispanidad, 10, 50009 Zaragoza, Spain; 3 Department of Physical Therapy, Occupational Therapy, Rehabilitation and Physical Medicine, Faculty of Health Sciences, Rey Juan Carlos University, Alcorcón, 28922 Madrid, Spain; 4 Motion Analysis, Biomechanics, Ergonomy and Motor Control Laboratory (LAMBECOM), Faculty of Health Sciences, Rey Juan Carlos University, Alcorcón, 28922 Madrid, Spain

**Keywords:** assessment, neurological disorders, psychometric properties, upper limb function, virtual reality

## Abstract

**Background:** Virtual reality (VR) is increasingly being explored as a tool for upper limb (UL) assessment in adults with neurological disorders. This review synthesizes the available evidence on VR-based UL assessments by analyzing their psychometric properties and potential role in clinical and research settings. **Methods:** This systematic review followed the Guideline for reporting systematic reviews of outcome measurement instruments (PRISMA-COSMIN) and was prospectively registered in PROSPERO. Data extraction was independently performed by two reviewers, with disagreements resolved by consensus or a third reviewer. Psychometric evidence was evaluated using the COnsensus-based Standards for the selection of health Measurement INstruments (COSMIN) methodology, including risk of bias assessment, evaluation and synthesis of measurement properties, and evidence grading according to GRADE. Additionally, the clinical application context of each VR assessment was examined, and a strengths, weaknesses, opportunities and threats analysis was conducted to explore factors affecting implementation and future development in neurorehabilitation. **Results:** Twenty VR-based UL assessments were identified. VR-Box and Blocks Test represented the largest body of evidence and was the only assessment implemented across all immersion levels, with promising findings in both stroke and Parkinson’s disease populations (Grade B recommendation). Immersive Action Research Arm Test demonstrated the strongest psychometric profile among the stroke sample (Grade B recommendation). Additionally, the Virtual Occupational Therapy Assistant and SaeboVR^®^ incorporated the most ecologically valid tasks, reflecting activities closer to daily life performance. Test–retest reliability and construct validity were the most frequently evaluated measurement properties. **Conclusions:** VR-based assessments represent promising tools for UL assessment. However, despite most systems receiving a Grade B recommendation, the supporting evidence remained low or very low certainty due to methodological shortcomings and incomplete psychometric evaluation. Therefore, more rigorous and methodologically robust research is needed to strengthen the evidence supporting their implementation in clinical practice as a complementary tool for UL assessment.

## 1. Introduction

Neurological disorders are among the leading causes of disability worldwide, generating substantial limitations in functional independence and quality of life [1]. Among the various impairments associated with these conditions, upper limb (UL) dysfunction is particularly common and disabling, significantly affecting the performance of activities of daily living (ADL) and social participation. This impairment is highly prevalent in individuals with stroke, Parkinson’s disease, multiple sclerosis, traumatic brain injury, and spinal cord injury, among other neurological conditions [2,3].

Accurate assessment of UL function is essential in neurological rehabilitation [4], as it allows the evaluation of the different components of functioning affected by neurological disorders within the framework of the International Classification of Functioning, Disability and Health (ICF) [5], supporting clinical decision-making, goal setting, and outcome evaluation. However, many conventional assessment tools exhibit limited ecological validity, rely on subjective scoring procedures, require expensive specialized equipment, or fail to evaluate clinically relevant aspects of UL performance, including movement quality, praxis, grasp patterns, and object interaction during functional activities [6,7].

Extended reality refers to a group of immersive technologies that include virtual reality (VR), augmented reality, and mixed reality, spanning the reality–virtuality continuum [8]. VR provides a fully computer-generated environment that replaces the user’s perception of the physical world. Augmented reality enhances the real environment by superimposing digital content. In contrast, mixed reality encompasses environments in which real and virtual elements are integrated to varying degrees while allowing users to interact with both the physical and digital worlds. In recent years, extended reality has attracted increasing interest in neurorehabilitation, offering new opportunities for clinical assessment and support more engaging and effective therapeutic interventions. In this context, VR has emerged as a promising tool [8,9]. VR enables users to interact with computer-generated environments through varying levels of immersion, commonly classified as immersive (high immersion and sense of presence), semi-immersive (partial immersion while preserving movement tracking and interaction with virtual content), and non-immersive systems (screen-based interaction using external devices such as controllers or buttons) [10,11].

From a patient-centred perspective, the successful implementation of VR in neurorehabilitation depends on multiple factors, including the type of hardware, the level of immersion and interaction, sociocultural and socioeconomic characteristics, technology acceptance, and the patient’s motor, cognitive, and sensory abilities. Furthermore, it is highly important to consider patients’ attitudes towards and acceptance of VR technologies. These factors can strongly influence user experience, engagement, and ultimately the effectiveness of the intervention [12]. In addition, the increasing adoption of VR technologies has accelerated the development of telerehabilitation, expanding access to neurorehabilitation services and enabling the delivery of personalized interventions beyond conventional clinical settings [13,14].

In the context of clinical assessment, VR enables standardized and controllable assessments within ecologically relevant environments [15]. Consequently, both virtualized adaptations of conventional UL measures and VR systems specifically designed for UL assessment have emerged. However, evidence regarding the psychometric properties and clinical utility of these virtual systems remains limited and heterogeneous. Previous reviews have focused on VR therapeutic effects, whereas a comprehensive synthesis evaluating the psychometric properties of VR-based UL assessment across neurological disorders, technological approaches, and levels of immersion remains lacking [9]. Consequently, a systematic evaluation of their measurement properties is crucial to determine their validity and readiness for clinical and research deployment.

The present review aims to systematically analyze the existing evidence on VR for UL assessment in adults with neurological disorders, evaluate its psychometric properties, and define its potential role in clinical and research settings. Secondary objectives are to identify the technological characteristics of the available systems, describe the UL functions and tasks assessed, and highlight current methodological limitations.

## 2. Methods

### 2.1. Design

This systematic review was conducted in accordance with the Guideline for reporting systematic reviews of outcome measurement instruments (PRISMA-COSMIN) (Supplementary Material Table S1) [16]. The review protocol was prospectively registered in the International Prospective Register of Systematic Reviews (PROSPERO) under registration number [CRD420251237279].

### 2.2. Search Strategy

This systematic review was conducted to identify and analyze validated virtual environments designed to assess UL function in adult patients with neurological conditions, with a specific focus on their psychometric properties.

The review question was formulated according to the Population, Intervention, Comparator, Outcome (PICO) framework as follows: in adult patients with neurological conditions (P), which validated virtual environments designed to assess UL function (I) are available, and what are their psychometric properties (O)? No comparator (C) is required, as this review aims to identify and evaluate assessment tools rather than compare interventions.

A comprehensive literature search was conducted in the following electronic databases: PubMed, Scopus, Web of Science, Science Direct, Cochrane, PEDro and OTseeker, covering all publications from database inception until 25 November 2025.

Combinations of keywords, including both controlled vocabulary (Medical Subject Headings [MeSH] and free-text terms), were used. Truncation and Boolean operators (AND, OR, NOT) were applied to combine the search terms appropriately.

The search strategy was structured around four key concepts:

1. UL function (“upper limb”, “upper extremity”, “hand”, “manual dexterity”).
2. VR technology (“virtual reality”, “VR”, “immersive”, “head-mounted display”, “HMD”).
3. Assessment instruments (“assessment”, “evaluation”, “scale”, “test”, “measurement”, “instrument”, “outcome measure”).
4. Psychometric properties measured (“validity”, “validation”, “reliability”, “psychometric”, “test–retest”, “responsiveness”, “measurement properties”).

The search strategy was adapted for each database according to its specific syntax and indexing system.

### 2.3. Study Selection

Studies were eligible if they met the following criteria: adult participants (≥18 years) with neurological disorders; empirical studies (including observational designs and diagnostic assessments) that evaluate the psychometric properties, feasibility or validity of UL functionality assessment instruments adapted to or designed for VR environments; studies published with no language or date of publication restrictions.

This systematic review excluded articles according to the following exclusion criteria: publications lacking full-text access, poster presentations, conference abstracts, symposium reports, or technical analyses without clinical relevance or application.

### 2.4. Data Collection

All records were imported into the reference management software “Rayyan AI” (Rayyan Systems Inc., Cambridge, MA, USA; web version), used by two independent reviewers (JBL and ÁAR) to remove duplicates and the screening process of titles and abstracts against the eligibility criteria. Full texts of potentially relevant articles were obtained and assessed independently by the same reviewers. Disagreements were resolved through discussion or by consulting a third reviewer (RCC). Inter-rater agreement was high, with only minor discrepancies, which were resolved by consensus.

Extracted information was collected using a standardized and piloted data extraction form to gather information about authors, year of publication, VR test or scale, type of VR, disease, sample size, study design, domains assessed linked to UL, ICF domains, type of praxis, estimated administration time, patient position, comparator measures, additional material used, level of satisfaction and adverse effects. When multiple studies validated the same scale, results were synthesized, and a narrative synthesis was conducted. Due to the anticipated heterogeneity in instruments, study designs, and populations, a meta-analysis was not initially proposed. Instead, the available evidence was synthesized narratively, with key findings presented in tabular format.

### 2.5. Evaluation of Selected Studies

To assess the psychometric robustness of existing UL assessment used in VR settings, we will apply the methodology proposed by the COnsensus-based Standards for the selection of health Measurement INstruments (COSMIN) initiative for systematic reviews of patient-reported outcome measures (PROMs) [17]. The evaluation will be structured across three steps:

Step 1: Assessment of risk of bias

We will evaluate the methodological quality of each included study using the COSMIN risk of bias checklist [17]. Each psychometric property assessed in a validation study (across the three COSMIN domains covering various measurement properties: reliability (internal consistency, reliability, and measurement error), validity (content validity, structural validity, hypothesis testing for construct validity, cross-cultural validity, and criterion validity), and responsiveness will be rated according to predefined criteria as “Very Good”, “Adequate”, “Doubtful”, or “Inadequate”, based on study design quality, statistical methods, and reporting.

The overall rating for the quality of each study for a measurement property will be determined by the lowest rating for any standard (“the worst score counts”). This step will allow for a transparent identification of potential limitations in the psychometric evidence of each instrument.

Step 2: Quality of psychometric properties measurement

Measurement properties will be extracted and evaluated according to the updated COSMIN criteria for assessing good measurement properties [17]. Each property will be rated as “+” (sufficient), “-” (insufficient), or “?” (indeterminate). This phase will allow us to assess the quality of each psychometric property analyzed.

Step 3: Summary of evidence and recommendation grade (GRADE)

In accordance with COSMIN and GRADE (Grading of Recommendations Assessment, Development and Evaluation) guidelines [17,18], we will synthesize the evidence for each measurement property across scales, considering the number of studies, consistency of findings, quality (risk of bias), and precision. Each instrument will be assigned an overall level of evidence (high, moderate, low, or very low) and a recommendation classification as: A (recommended): sufficient evidence of adequate measurement properties; B (potentially recommended): promising but incomplete evidence; and C (not recommended): evidence showing inadequate psychometric performance.

Furthermore, the clinical application contexts of the included VR assessments will be examined, and a Strengths, Weaknesses, Opportunities, and Threats (SWOT) analysis will be conducted to evaluate factors influencing their implementation and future development in neurorehabilitation.

## 3. Results

### 3.1. Study Selection

**Figure 1:**
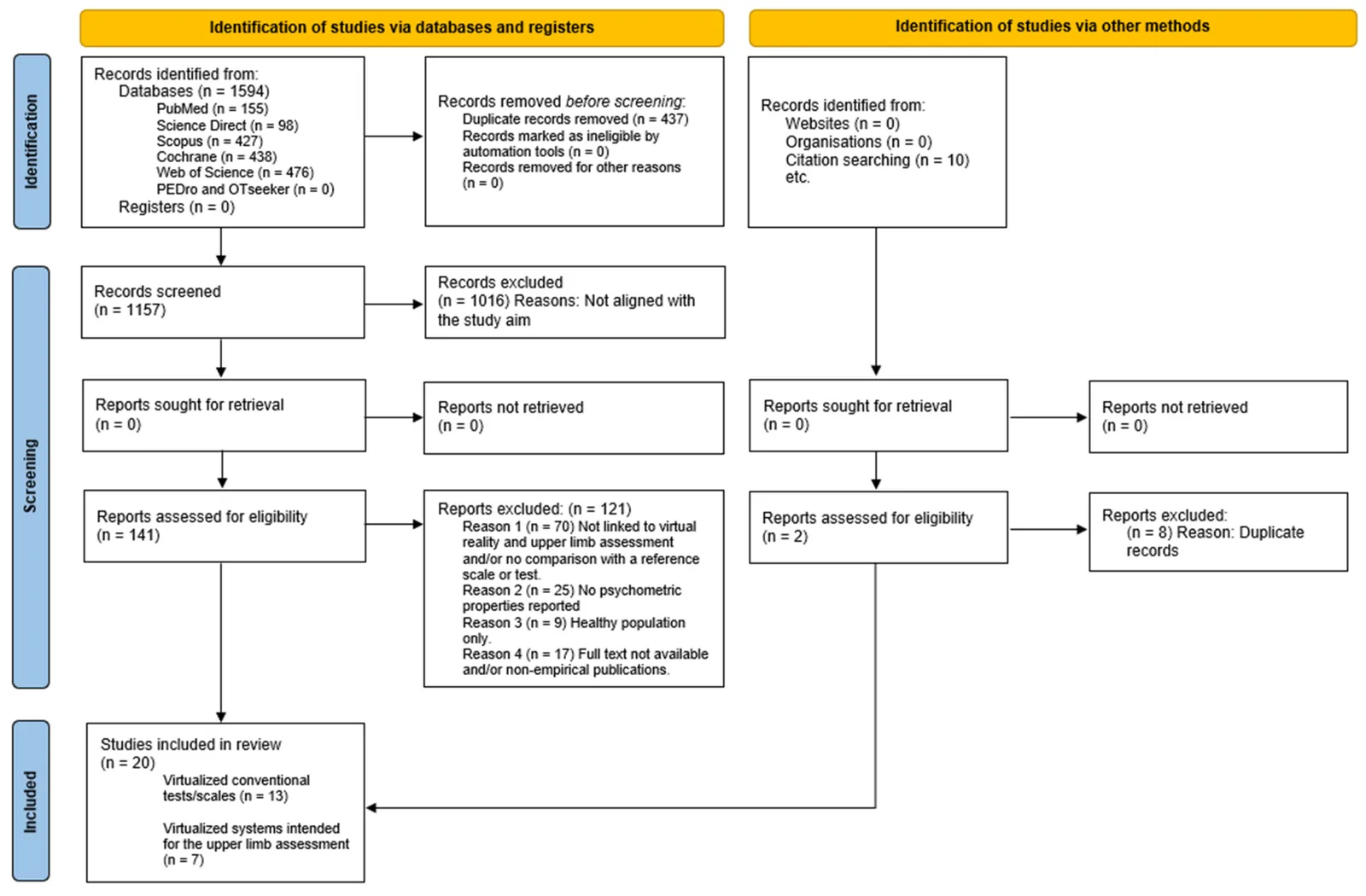
PRISMA flow chart.

The database search yielded 1594 records, with 1157 remaining after duplicate removal. Following title and abstract screening, 141 full-text articles were assessed for eligibility. Ultimately, 20 virtual assessments were included in this review (see PRISMA flow diagram in Figure 1). To facilitate analysis, two subgroups were established: virtualized conventional tests/scales (*n* = 13) and virtualized systems intended for UL assessment (*n* = 7).

### 3.2. Characteristics of Included Assessment Instruments

Twenty virtual assessments were included in this review [19,20,21,22,23,24,25,26,27,28,29,30,31,32,33,34,35,36,37,38]. All of them were published between 2010 and 2025. The articles were published in these countries Belgium (*n* = 3), South Korea (*n* = 3), Spain (*n* = 3), Switzerland (*n* = 3), United States (*n* = 3), China (*n* = 1), Germany (*n* = 1), Iran (*n* = 1), Sweden (*n* = 1), Turkey (*n* = 1). Table 1 summarizes the main characteristics of both groups: virtualized conventional tests/scales and virtualized systems intended for UL assessments. Furthermore, subgroups were established according to the specific type of VR (level of immersion) of each system. The specifications of the included VR assessments analyzed are detailed below.

Regarding the study populations, the included studies evaluated adults with neurological disorders. Stroke was the most represented condition (*n* = 393), with a sex distribution of 62.34% male and 37.66% female [21,22,23,24,25,26,28,29,30,33,34,35,37,38]. Among these, 306 participants were included in studies reporting mean ± standard deviation, with mean age values ranging from 55.63 to 67.88 years [21,22,23,24,25,26,28,29,32,35,37]. The remaining 87 participants reported median age, with values ranging from 57 to 69 years and reported age ranges extending from 25 to 87 years [30,33,34,38]. Parkinson’s disease studies included 63 participants aged 58.8–74.4 years, of whom 67.9% were male and 32.1% were female [19,20,36]. Multiple sclerosis was represented by 31 participants with a mean age of 56 years and a sex distribution of 51.61% male and 48.39% female [31]. Cervical spinal cord injury and other neurological disorders (Guillain-Barré syndrome and sequelae of infectious meningoencephalitis) comprised 12 participants with a mean age of 22.66 years, with a sex distribution of 75% male and 25% female [27].

The severity of neurological impairment among the included populations was commonly described using disease-specific clinical measures, although explicit eligibility thresholds were inconsistently reported across studies. In stroke populations, the motor impairment was commonly assessed using the Fugl-Meyer Assessment Upper Extremity [39] with participants ranging from severe to mild deficits. Additional eligibility criteria included transferring at least one cube during the conventional Box and Blocks Test (BBT) [23,40], moving at least three blocks per minute [22], and demonstrating a minimum of 10° active wrist extension in the affected UL [38]. Cognitive screening was incorporated using the Mini-Mental State Examination (MMSE) (≥24 points) [26,29,38,41] or the Montreal Cognitive Assessment (MoCA) (≥21) [23,42]. Although other clinical measures, including the Action Research Arm Test (ARAT) [43], Wolf Motor Function Test [44], Brunnstrom stages [45], and National Institutes of Health Stroke Scale [46], were frequently reported, they were generally used to characterize participants or evaluate validity rather than to define eligibility criteria. In Parkinson’s disease, participants generally presented mild-to-moderate disease severity according to the Hoehn and Yahr (stages I-IV) [19,20,36,47], while cognitive screening was performed using MMSE ≥ 24–26 or MoCA ≥ 24 [20,36]. Although motor severity was frequently characterized using the Movement Disorders Society Unified Parkinson’s Disease Rating Scale (MDS-UPDRS III) [48], no specific UPDRS-based eligibility thresholds were identified [19].

In multiple sclerosis, participant selection was restricted to individuals with mild-to-moderate UL dysfunction and without severe cognitive, sensory, or motor deficits that could interfere with task performance. Although disability was characterized using the Expanded Disability Status Scale (EDSS) [49], no EDSS-based eligibility thresholds were reported [31]. Similarly, in cervical spinal cord injury and other neurological conditions, no severity-related clinical thresholds were used for participant selection. Eligibility was based on the ability to complete both the conventional and virtual BBT tasks, whereas neurological status was described using the American Spinal Injury Association Impairment Scale, neurological level of injury [50], and Motor Index Score [51], no specific AIS grades, injury levels, or Motor Index Score ranges, without applying specific ranges or grades as inclusion criteria [27].

Finally, beyond participant sociodemographic and clinical characteristics, several studies also reported complementary outcomes related to user interaction with the VR systems. Notably, praxis was not formally assessed in any of the VR systems included, although some tasks incorporated sequential or goal-directed actions [20,21,24,25,30]. User experience was evaluated in a limited number of studies, with generally favourable findings regarding satisfaction, usability, enjoyment, or motivation [20,21,22,23,24,25,31]. Adverse effects were only evaluated by one study and reported no adverse symptoms [32]. Overall, these outcomes were inconsistently evaluated and predominantly not reported across studies.

#### 3.2.1. Virtualized Conventional Tests/Scales

VR Finger Tapping test (VR-FTT)

The finger tapping test (FTT) is an immersive VR system used to assess motor function, specifically identifying hypokinetic and arrhythmic profiles. It consisted of repetitive flexion–extension movements of the index finger at the metacarpophalangeal joint while the subject was seated in a relaxed position, with the arms resting on a table and the elbow at approximately 90–100° [52].

Participants wore an HMD that provided a first-person perspective within a minimal virtual environment, including an avatar, a chair, and a table. They were instructed to perform finger tapping at different self-selected rhythms (fast, normal, and slow). UL movements were captured and reproduced in real time, allowing users to perceive a realistic three-dimensional representation of their own actions [19].

Virtual Reality Box and Blocks Test (VR-BBT)

The VR-BBT was based on the conventional BBT, a standardized, performance-based measure of unilateral gross manual dexterity. It assesses the number of blocks an individual can transfer, one at a time, from one compartment of a box to another within 60 s, providing a quantitative outcome of UL function [40].

Different versions of the VR-BBT have been developed, varying according to the authors and the technological systems employed. These implementations can be broadly classified into immersive, semi-immersive, and non-immersive VR modalities.

Immersive VR systems fully occlude the real environment and typically rely on head-mounted displays (HMD). Some studies relied primarily on hand-tracking solutions, such as the combination of the Oculus Rift^®^ (Oculus VR, Menlo Park, CA, USA) with the Leap Motion Controller^®^ (Leap Motion, Inc., San Francisco, CA, USA) [20], whereas others implemented a controller-based interaction [21,24]. In contrast, some authors sought to enhance somatosensory feedback by integrating the Omega.7^®^ (Force Dimension, Nyon, Switzerland) haptic interface with the HMD [28]. More recent approaches have also explored differences between the controller-based interaction with hand-tracking modalities [23].

Semi-immersive systems, which typically involve interaction with virtual environments displayed on external screens while maintaining partial awareness of the real world, have also been explored using the Microsoft Kinect^®^ (Microsoft Corporation, Redmond, WA, USA) [26] to capture UL movements, while others implemented the system using the Leap Motion Controller^®^ for fine motor tracking [27].

In contrast, non-immersive VR systems rely on conventional displays and external devices without isolating the user from the real environment. Within this category, a robotic VR assessment based on the BBT, using the Hand Wrist Assistive Rehabilitation Device (HWARD) in conjunction with a standard monitor, was developed. However, this version differed from the conventional test by focusing on finger grasp and release without requiring the shoulder movements needed to transfer blocks over a partition, limiting the maximum rate of block presentation, and extending the assessment duration from 1 to 3 min [28].

Overall, these variations highlight the technological heterogeneity of VR-BBT implementations and suggest that the level of immersion and type of interaction device may influence both user experience and assessment outcomes.

Virtual Peg Insertion Test (VPIT)

The VPIT was developed based on the conventional Nine Hole Peg Test (NHPT). The NHPT is a standardized, performance-based measure of fine manual dexterity, in which individuals are required to place and remove nine pegs into holes on a board as quickly as possible, with completion time used as the primary outcome [53].

All identified VPIT implementations correspond to non-immersive VR systems and share a highly similar technological setup. Specifically, consistently employed a monitor in combination with haptic-feedback devices such as the Phantom Omni^®^ (SensAble Technologies Inc., Woburn, MA, USA) and/or Geomagic Touch^®^ (3D Systems, Rock Hill, SC, USA). Despite the shared hardware configuration, variability across studies arises from methodological heterogeneity in its implementation [29,30,31].

Action Research Arm Test Virtual Reality (ARAT-VR)

The ARAT-VR was based on the conventional ARAT, a standardized, performance-based measure of UL function consisting of 19 items subdivided into four subtests (grasp, grip, pinch, and gross movement), which assess the ability to perform task-oriented functional movements [43].

The ARAT-VR was implemented with the Oculus^®^ (Oculus VR, Menlo Park, CA, USA) Quest 2 headset featuring infrared hand-tracking, connected to a computer for real-time monitoring. It included 13 of the 19 original ARAT items across four subtests. Six items were excluded due to hand-tracking limitations, which prevented accurate assessment of object weight and fine manipulations. As a result, only cubes and 1.5 cm marbles were used, and the behind-the-head task was removed. A longer time threshold (10 s) was set for the maximum score based on pretest findings. Items were scored from 0 to 3, and total scores ranged from 0 to 39, with higher scores indicating better UL function [25].

#### 3.2.2. Virtualized Systems Intended for UL Assessment

Virtual Environment Technique (VET)

This VR system is classified as an immersive VR system with limited interaction, as it uses an HMD, whose specific model is not specified, together with an eye bandage to fully occlude the real environment. The setup includes a custom-built Virtual Reality Upper Extremity Tester (VRUPT), joint encoders for angle measurement, and a computer system.

The virtual environment is simple and task-oriented, consisting of geometric elements such as lines (for angle matching) and cylinders (for reaching tasks), designed to assess proprioception through measures like angular error and execution time [32].

Virtual Occupational Therapy Assistant (VOTA)

VOTA is a semi-immersive virtual system designed to assess and train UL function in stroke patients through the performance of ADL across different rehabilitation settings. It uses a Kinect^®^ (Microsoft Corporation, Redmond, WA, USA) sensor, kinematic pose estimation algorithms, and game engine technology to capture and reproduce patient movements within an interactive virtual environment. The virtual environment allowed manipulation of object position and task demands to target specific joint movements and ranges of motion. Compensatory strategies, such as trunk movement, were minimized by restricting avatar motion to the affected limb, ensuring that recorded performance reflected true motor capacity [33].

SaeboVR^®^

SaeboVR^®^ is a semi-immersive system that combines a commercial SaeboGlove^®^ Orthosis with motion sensors to track finger joint angles during grasp–release tasks within a virtual environment. Combined with Microsoft Kinect^®^ data and kinematic algorithms, the system enabled real-time estimation of UL joint movements and reconstruction of the patient’s avatar.

The integration of the glove extended the SaeboVR^®^ system by enabling active grasp–release interactions, requiring both hand positioning and specific finger movements. Tasks were decomposed into subtasks with defined start and end points, allowing automatic segmentation and analysis of movement quality and speed. The system generated kinematic performance metrics based on functional activities, while providing visual and auditory feedback [34].

Virtual target-to-target pointing task

This VR system consisted of a semi-immersive workbench with a 3D display viewed through stereoscopic glasses, creating the perception of depth via synchronized image projection.

Kinematic data were captured using the PHANTOM OmniTM^®^ (SensAble Technologies Inc., Woburn, MA, USA) haptic device, a stylus-based device with six degrees of freedom that allowed free movement within the virtual workspace. The system provided both visual and haptic feedback, generating the illusion of physically interacting with virtual objects [35].

Computerized exergaming system

This semi-immersive system was developed using the Microsoft Kinect^®^ as a motion capture sensor, and a monitor positioned in front of the participant. Participants controlled an avatar to perform reaching and tracking tasks involving ball-shaped targets, designed to challenge uni–bimanual and symmetric–asymmetric movements. The environment was optimized for cognitive and visuospatial impairments, and compensatory movements were not considered.

Reaching tasks required touching appearing targets with visual and auditory feedback, promoting movements in multiple directions. Tracking tasks involved following predefined paths after activating the target, also with feedback, and were performed in both unimanual and bimanual modes [36].

Multi-joint arm exoskeleton

This non-immersive VR system integrated a rehabilitation exoskeleton (Armeo^®^ Spring (Hocoma AG, Volketswil, Switzerland), Hocoma) equipped with sensors for shoulder, elbow, wrist, and grip force, which was used to provide gravity support and record UL kinematics. The device was individually adjusted to each patient and standardized in a neutral starting position, allowing accurate and comparable measurements. The system integrated assessment and training within the same VR environment, reducing variability and enabling standardized evaluation [37].

Upper Extremity Smart Exercises-Innovate Treatment (USE-IT^®^)

The non-immersive system USE-IT^®^ comprised a game-based system designed for objective assessment and rehabilitation of UL function using a touchscreen interface. Evaluation was performed through the Balloon-Popping Game, where patients reached and touched targets appearing at different screen locations, generating performance data based on accuracy and time.

The system produced quantitative outcomes by comparing patient performance with normative data from healthy individuals, generating maps with a colour-coded scale to represent reaching difficulty across different areas. Scores were calculated based on the percentage of each colour zone, ranging from 100 to 500 points, with higher scores indicating better UL function [38].

### 3.3. COSMIN-Based Evaluation of Measurement Properties

Step 1. Methodological quality assessment (risk of bias assessment): The methodological quality of the included studies was assessed using the COSMIN risk of bias checklist. The studies analyzed were characterized by an absence of PROM development, content validity, structural validity, internal consistency, cross-cultural validity and responsiveness. Content validity was investigated only for the ARAT-VR [25] and the immersive VR-BBT hand-tracking and controller version [23]. Responsiveness, defined by COSMIN as the ability of a measurement instrument to detect change over time in the construct to be measured [17], was evaluated only for the VPIT in multiple sclerosis [31].

Reliability was investigated in ten studies [19,20,21,23,24,25,29,30,32,36], predominantly using intraclass correlation coefficients (ICC), although one study used Pearson’s correlation coefficients [32] to assess test–retest reliability. Measurement error-related indices were reported in seven studies [20,21,23,25,29,30,36], including the standard error of measurement (SEM), minimal detectable change (MDC), smallest detectable change (SDC), and systematic error, depending on the study. Criterion validity was established using the corresponding conventional version upon which each VR system was originally based and was investigated in eleven studies of virtualized conventional assessments [20,21,22,23,24,25,26,27,29,30,31], by comparing the VR-based assessment with the corresponding conventional test on which it was based. Construct validity was the most frequently investigated measurement property, with associations between VR-derived outcomes and established clinical measures examined in eighteen studies [20,21,22,23,24,25,26,27,28,29,30,31,33,34,35,36,37,38].

According to the COSMIN risk of bias assessment, the methodological quality of the available evidence was predominantly rated as “Doubtful”. Content validity was only evaluated by two studies and was rated as “Doubtful” [23,25]. Reliability was evaluated by ten studies and was predominantly rated as “Doubtful” [20,21,23,24,25,29,30,36], whereas the methodological approaches used to evaluate reliability for the VR-FTT [19] and VET [32] were rated as “Inadequate”. Measurement error was evaluated by seven studies and was also rated as “Doubtful” [20,21,23,25,29,30,36]. Criterion validity was evaluated by eleven studies and was predominantly rated as “Doubtful” [20,21,22,23,24,26,27,29,30,31], and the ARAT-VR [25] received an “Adequate” rating. Similarly, hypothesis testing for construct validity was evaluated in eighteen studies and predominantly rated as “Doubtful” [20,21,22,23,24,26,27,28,29,30,31,33,34,35,36,37,38], with the ARAT-VR [25] receiving an “Adequate” rating. Responsiveness, assessed only for the VPIT in multiple sclerosis [31], was rated as “Doubtful”.

Step 2. Quality of Psychometric Properties Measurement: Each measurement property was evaluated using predefined criteria and classified according to the COSMIN criteria for assessing good measurement properties checklist.

Although two studies [23,25] conducted content validity analyses, these were deemed incomplete or methodologically insufficient. Therefore, they were rated as “?”. For the remaining studies, no evidence was reported regarding content validity, structural validity, internal consistency, or cross-cultural validity. Consequently, these were rated as “?”.

Reliability was rated as sufficient “+” for the immersive VR-BBT evaluated in Parkinson’s disease [20] and stroke [21,23,24], the ARAT-VR [25], and the semi-immersive computerized exergaming assessment system evaluated in Parkinson’s disease [36]. In contrast, reliability was rated as insufficient “-“ for the VR-FTT [19] and the VPIT in stroke [29,30], while it remained indeterminate “?” for the remaining assessments. Measurement error was rated as “?” across all assessments, as the available evidence did not permit a definitive classification according to the predefined criteria for this property.

Criterion validity showed “+” ratings for the immersive VR-BBT [21,22] and the hand-tracking version [23], and the ARAT-VR [25], and the semi-immersive VR-BBT [26]. Conversely, criterion validity was rated as insufficient “-” for the immersive VR-BBT evaluated in Parkinson’s disease [20], the controller-based VR-BBT [23], both physical and non-physical-interaction of VR-BBT versions [31], and the VPIT in stroke and multiple sclerosis [29,30,31]. Criterion validity remained indeterminate “?” for the remaining assessments.

Hypothesis testing for construct validity was rated as sufficient “+” only for the ARAT-VR [25]. Among the remaining studies in which construct validity was investigated [20,21,22,23,24,26,27,28,29,30,31,33,34,35,36,37,38], the results were rated as indeterminate “?”, because hypotheses were either not predefined or, when formulated, were not specified using explicit and quantifiable criteria to determine whether they had been confirmed. No evidence on construct validity was available for the remaining assessments [19,32].

Finally, responsiveness was investigated only for the VPIT in multiple sclerosis [31] and was rated as indeterminate “?”.

Step 3. Grading the Quality of Evidence (GRADE Approach): Using the GRADE framework, the overall quality of evidence per measurement property was determined by considering risk of bias, inconsistency, imprecision, and indirectness. Grades follow COSMIN categories: A = recommended; B = potentially recommended; C = not recommended. Furthermore, the level of evidence from each assessment was defined from high to very low. Table 2 summarizes the GRADE-based overall recommendations for each instrument, integrating the quality, consistency, and completeness of the available psychometric evidence to support comparative interpretation and decision-making.

Overall, most VR-based UL assessments received a Grade B recommendation, although the level of evidence remained predominantly low or very low. Virtualized conventional tests generally demonstrated a more consistent body of psychometric evidence than VR systems intended for the UL assessment. Stroke represented the neurological condition with the strongest body of evidence, with the immersive ARAT-VR [25] demonstrating the most favourable overall psychometric profile, while multiple versions of the VR-BBT consistently achieved Grade B recommendations across different immersion levels [20,21,22,23,24,26,27,28]. The VR-FTT also demonstrated a promising psychometric profile despite being supported by very low-certainty evidence [19]. By contrast, the VPIT was the only assessment that consistently received a Grade C recommendation across both stroke and multiple sclerosis populations [29,30,31]. Furthermore, VR systems intended for the UL assessment were generally supported by Grade B recommendations, with SaeboVR^®^ showing the strongest evidence (low certainty) [34]. In contrast, all remaining systems were supported by very low-certainty evidence due to methodological shortcomings and insufficient psychometric validation [19,32,33,35,36,37,38].

### 3.4. Clinical Application Contexts

Few VR assessments of UL functionality have reported validations in specific clinical populations related to neurorehabilitation, including adults with neurological conditions, indicating a critical gap in scale generalizability across clinical contexts.

The reviewed VR-based assessment tests demonstrate substantial heterogeneity in their application contexts, largely reflecting differences in system design and the characteristics of the validation populations.

Stroke populations represented the most extensively studied clinical group. Regarding the identified virtualized conventional tests, studies included the VR-BBT: immersive [21,22,24], semi-immersive [26] and non-immersive versions [28]. Additionally, the immersive ARAT-VR [25] and non-immersive applications of the VPIT [29,30] were reported.

Regarding the subgroup related to virtualized systems intended for UL assessment, several systems were identified in stroke, including the immersive VET [32], the semi-immersive VOTA [33], SaeboVR^®^ [34], the semi-immersive Virtual Target-to-target pointing task [35], the non-immersive Multi-joint Arm Exoskeleton System [37], and the non-immersive USE-IT^®^ system [38].

In Parkinson’s disease, the evaluated systems included the immersive VR-FTT [19] and VR-BBT [20], and a semi-immersive computerized exergaming system [36].

In multiple sclerosis, evidence was limited to a single system, namely the non-immersive VPIT [30], highlighting the scarcity of validation studies in this population.

Finally, in cervical spinal cord injury and other neurological conditions (Guillain-Barré syndrome and sequelae of infectious meningoencephalitis), only a semi-immersive VR-BBT system was identified [27].

Based on the current state of the art, a SWOT (Strengths, Weaknesses, Opportunities, and Threats) analysis has been developed to synthesize the key considerations regarding VR assessment of UL functionality and its possible development in the context of neurorehabilitation in Table 3.

## 4. Discussion

The aim of this review was to identify existing VR assessments of UL functionality, examine their psychometric quality, and evaluate their applicability in neurorehabilitation. Our findings show that, despite the growing clinical relevance of VR technologies, validated UL assessments with VR environments specifically in adults with neurological impairments remain scarce. The identified systems could broadly be classified into two categories: virtualized versions of conventional tests/scales and virtualized systems intended for UL assessment. Although both approaches aim to quantify UL performance, they differ substantially in technological requirements, ecological validity, and the psychometric evidence currently available.

Although VR-based UL assessments increasingly incorporate functional interactions, most remain centred on reaching, transport, and object displacement tasks. From an ICF perspective, most systems assessed the “Activities and Participation” domain through standardized UL tasks, whereas a smaller number additionally quantified “Body functions” using kinematic, kinetic, or sensorimotor metrics. However, participation in real-life contexts was not directly assessed. Although VOTA [33] and SaeboVR^®^ [34] incorporated instrumental ADL-inspired activities and ARAT-VR included more functionally relevant object manipulation tasks, most platforms relied on highly structured assessments that only scarcely or partially reflected the complexity of everyday performance [20,21,22,23,27,29,30,31]. Consequently, important aspects of UL functioning, such as grasp typology, in-hand manipulation, motor planning, praxis, movement quality, and compensatory strategies, remain largely unexplored despite their relevance for occupational performance. This observation is consistent with the previous literature highlighting the distinction between motor capacity and real-world performance, suggesting that the ecological validity and functional transferability of assessment outcomes remain uncertain [54].

Across studies, participants generally presented with mild to moderate UL impairment and preserved cognitive function sufficient to understand instructions and complete VR-based tasks (MMSE ≥ 24 points and MoCA ≥ 21) [23,26,29,38]. However, none of the included studies specifically examined whether age-related differences in cognitive function influenced performance using VR. Common inclusion criteria included residual active UL mobility, preserved manual dexterity, and the ability to perform reaching or manipulation tasks within virtual environments, whereas severe cognitive, visual, or psychiatric disorders and any condition limiting adequate interaction with the technology were frequently excluded. These findings are consistent with previous reviews, suggesting that the psychometric properties reported for these systems may not be readily generalizable to neurological populations with more severe levels of impairment [55]. In this context, performance in VR-based assessments may reflect not only UL function but also the ability to interact effectively with controllers, hand-tracking systems, haptic devices, sensors, or HMD.

Another important consideration is the context of application. Although VR offers clear potential for automated and remote assessment, the included systems were validated under supervised clinical or laboratory conditions. Therefore, their measurement properties may not be directly transferable to home-based or telerehabilitation settings, where variations in equipment setup, environmental conditions, technical support, and supervision could influence performance. Telerehabilitation is increasingly being implemented in neurorehabilitation and has demonstrated promising results for intervention delivery [56,57]. However, evidence supporting the use of VR-based assessments in remote settings remains limited. Further research is needed to determine whether the validity, reliability, usability, and safety of these assessments can be maintained remotely.

The external validity of the findings reported in this review should be interpreted considering the substantial methodological and technological heterogeneity across studies. Differences in neurological populations, VR systems, hardware configurations, and assessment tasks limit comparability between results and hinder standardization. Similar concerns have been highlighted in previous reviews, which identified technological variability, limited longitudinal evidence, and the lack of large-scale validation studies as important barriers to clinical implementation. Nevertheless, patients generally exhibit positive attitudes toward these tools, suggesting that their acceptance may facilitate their successful implementation in neurorehabilitation, provided that individual patient characteristics and technological requirements are appropriately considered [12,14]. Furthermore, VR-based assessments offer several potential advantages, including enhanced user motivation, engagement, adherence, reduced dependence on dedicated physical equipment, and the possibility of automated or remote evaluation [9,58].

Economic and implementation factors may further influence the adoption of VR-based assessments. The systems identified ranged from low-cost solutions to highly specialized platforms, resulting in substantial variability in implementation costs. Beyond hardware acquisition, factors such as maintenance, technical support, clinician training, scalability, and long-term sustainability should also be considered. Furthermore, integrating VR-based assessments into telerehabilitation pathways may improve accessibility while reducing travel demands and associated indirect costs [59]. However, evidence regarding the economic impact of VR-based assessment remains scarce, highlighting the need for future cost-effectiveness and implementation studies.

The European recommendations for Clinical Assessment of UL in Neurorehabilitation (CAULIN) constitute an expert-based framework developed to guide the selection of UL outcome measures in neurorehabilitation. According to these recommendations, assessment tools should demonstrate validity, reliability, responsiveness, clinical utility, and feasibility before being adopted in neurorehabilitation practice [4]. However, the psychometric evidence identified in this review remains incomplete. Among the included studies, test–retest reliability [19,20,21,23,24,25,29,30,32,36] and construct validity [20,21,22,23,24,25,26,27,28,29,30,31,33,34,35,36,37,38] were the most frequently investigated psychometric properties. This predominance may reflect the early developmental stage of many VR-based UL assessments, as these properties are typically the most accessible and clinically relevant during initial validation phases. However, the overall strength of the evidence remains limited by methodological shortcomings, including small sample sizes, the absence of sample size calculations, and insufficient hypothesis testing according to COSMIN recommendations.

Other measurement properties were only sparsely investigated. Measurement error was occasionally assessed using Standard Error of Measurement, although these parameters were not always presented explicitly [20,21,23,25,29,30,36]. Responsiveness was longitudinally evaluated only by one study [31], and content validity was limited mainly to expert judgments concerning task equivalence or difficulty of the virtual tasks [23,25], without a comprehensive evaluation of relevance, comprehensiveness, and comprehensibility involving both experts and patients. Furthermore, the included studies did not perform formal evaluations of structural validity, internal consistency, or cross-cultural validity. The lack of cross-cultural validity may limit the applicability, comparability, and replication of VR-based assessments across different countries, healthcare settings, and patient populations, as differences in language, familiarity with technology, healthcare practices, and sociocultural factors may influence patient interaction with virtual tasks [60]. These findings are in line with previous reviews, which reported that psychometric validation of VR-based assessments remains incomplete and is mainly restricted to reliability and validity analyses, despite the increasing use of these technologies [13,54].

Despite most assessments being classified as Grade B, the certainty of evidence remained predominantly low or very low. This apparent discrepancy reflects important limitations across the available literature, including small sample sizes, a lack of sample size calculations, risk of bias, other methodological shortcomings, and the incomplete evaluation of key COSMIN measurement properties. The more favourable psychometric evidence observed for virtualized conventional tests should therefore be interpreted with caution, as it primarily reflects that these systems have undergone broader COSMIN-based psychometric testing, whereas evidence for several key measurement properties remains unexplored for many VR systems intended for UL assessment, resulting in greater uncertainty regarding their measurement quality. Within the former group, the immersive VR-BBT [20,21,23] and ARAT-VR [25] demonstrated the most favourable overall psychometric profiles, whereas the VPIT [29,30,31] consistently received Grade C recommendations due to methodological limitations and incomplete psychometric evidence. Among VR systems intended for UL assessment, all were classified as Grade B because several key measurement properties remain unevaluated, precluding a definitive recommendation. However, SaeboVR^®^ [34] was the only system supported by low-certainty evidence. Consequently, the current evidence remains insufficient to determine whether either approach provides superior measurement performance, consistent with previous reviews highlighting the need for more comprehensive psychometric validation before these technologies can be considered robust assessment tools for clinical practice [9].

Future research should focus on validating these technologies in larger and more representative adult neurological populations, as many previous studies have primarily been conducted in healthy individuals [61,62,63,64]. Standardized protocols and more comprehensive psychometric evaluations according to COSMIN recommendations, including cross-cultural adaptation and validation, are needed to strengthen their clinical applicability, methodological quality and improve the comparability, reproducibility, and generalizability of VR-based assessments across different healthcare and cultural contexts [60]. Furthermore, workload, system usability, and potential adverse effects associated with VR use, such as cybersickness [65], visual discomfort, and fatigue, remain insufficiently explored and should be systematically investigated. Additional evidence is also required to determine whether VR-based assessments can maintain their psychometric performance when implemented in remote or telerehabilitation contexts [57,66]. Finally, future studies should also consider implementation-related factors, including hardware and software costs, maintenance requirements, technical support, and accessibility across different healthcare settings. Importantly, VR technologies encompass a wide spectrum of solutions, ranging from low-cost consumer devices to highly specialized professional platforms, which may allow adaptation to different clinical needs and economic contexts while facilitating broader implementation in neurorehabilitation practice [59,67].

This systematic review presents several methodological limitations. First, a meta-analysis was not performed due to the considerable heterogeneity across the included studies in terms of assessment systems, study designs, outcome measures, and neurological populations. Second, the search strategy was restricted to the selected databases, which may have resulted in the omission of relevant studies indexed in other sources. In addition, publication bias cannot be excluded, as studies reporting positive or significant findings are more likely to be published than studies with negative or non-significant results. Another important limitation is the absence or limited availability of psychometric evidence in neurological populations, particularly regarding content validity, structural validity, internal consistency, and cross-cultural validity, measurement error and responsiveness, according to COSMIN. Finally, the findings of this review cannot be generalized to other populations, technologies, or assessment scales that were not specifically evaluated in the included studies.

## 5. Conclusions

The available evidence suggests that VR-based systems are promising tools for UL assessment in adults with neurological disorders, particularly in stroke populations, where most of the current psychometric evidence is concentrated. According to the COSMIN-based evaluation, all identified systems were supported by low or very low certainty of evidence, mainly due to methodological limitations, small sample sizes, risk of bias, and the incomplete evaluation of key measurement properties, particularly content validity, structural validity, internal consistency, cross-cultural validity, responsiveness, and measurement error. Beyond conventional scales, VR has the potential to provide objective and automated assessment through kinematic and kinetic metrics while offering more engaging and context-oriented evaluation environments. Most systems focused on assessing UL performance at the activity level and, to a lesser extent, body functions, whereas participation in meaningful real-life contexts was rarely addressed.

Virtualized conventional tests currently provide the largest body of psychometric evidence for UL assessment according to COSMIN requirements. Among the available tools, immersive ARAT-VR demonstrated the most favourable psychometric profile in stroke populations (Grade B; low-certainty evidence), whereas VR-BBT adaptations provided the most evidence and were designed across all levels of immersion. Within these adaptations, the immersive VR-BBT showed the most promising findings in Parkinson’s disease and stroke (Grade B; low-certainty evidence). Among VR systems intended for UL assessment, SaeboVR^®^ achieved the most favourable level of evidence (Grade B; low-certainty evidence) and incorporated assessment tasks based on ADL. Evidence for multiple sclerosis, cervical spinal cord injury, and other neurological conditions remained highly limited, as each relied on a single study with very low-certainty evidence. Despite the methodological limitations of the available evidence, future research incorporating more rigorous psychometric validation could help strengthen the evidence base and support a more standardized implementation of these VR systems as complementary tools for upper limb assessment.

## Figures and Tables

**Table 1:** Main characteristics of the virtual tests identified.

**Virtualized Conventional Tests/Scales**								
**Type of VR**	**VR Test/Scale**	**Disease**	**Authors (year)/Country**	Sample Size/Mean Age ± SD * (M/F)	**Study Design**	**Domains Assessed Linked to UL/ICF Domain**	**Estimated Administration Time/Patient Position**	**Comparator Measures/Other Scales (if Applied)**
Immersive	VR-FTT	Parkinson’s disease	Sanmartín et al. (2013)/Spain [19]	10 patients/67.10 ± 2.92 years	Observational, cross-sectional, and validation study	Rhythmic motor execution/body functions	Not estimated time reported/seated position	Conventional FTT
Immersive	VR-BBT	Parkinson’s disease	Oña et al. (2020)/Spain [20]	*n* = 20 patients/74.35 ± 0.94 years (17M/3F)	Cross-sectional study	Gross dexterity and UL coordination/activities and participation	8 min estimated (1 min per hand)/seated position	Conventional BBT and Hoehn and Yahr Scale/ satisfaction and usability Likert questionnaire
Immersive	VR-BBT	Stroke	Everard et al. (2022)/ Belgium [21]	*n* = 22 patients/64 ± 10.9 years (17M/5F)	Cross-sectional study	Gross dexterity, arm kinematics, usability/activities and participation	6–8 total minutes (3 repetitions × 1 min per hand)/seated position	Conventional BBT/SUS questionnaire
Immersive	VR-BBT	Stroke	Dong et al. (2023)/China [22]	*n* = 16 patients/67.88 ± 10.93 years (11M/5F)	Observational pilot study	Gross manual dexterity, movement smoothness, efficiency and grasping/activities and participation	1 min test per hand/seated position	Conventional BBT, ARAT, FMA-UE, MMSE, Brunnstrom Stage/7-item questionnaire adapted from IMI
Immersive	VR-BBT	Stroke	Everard et al. (2024)/Belgium [23]	*n* = 21 patients/65 ± 7.2 years (15M/6F)	Cross-sectional study	Manual dexterity, kinematics, system usability/activities and participation	3 repetitions × 1 min per hand/seated position	Conventional BBT, CAT-FM/SUS questionnaire
Immersive	VR-BBT (Physical interaction version and no physical interaction version)	Stroke	Yun et al. (2025)/South Korea [24]	*n* = 31 patients/56 ± 19.5 years (16M/15F)	Observational study of clinometric/psychometric evaluation	UL sensorimotor and manipulation function and grip force control/body functions and activities and participation	Not estimated time reported/seated position	Conventional BBT, NHPT/SUS questionnaire
Immersive	ARAT-VR	Stroke	Burton et al. (2022)/Belgium [25]	*n* = 30 patients/59.8 ± 10.87 years (22M/8F)	Multicentric observational study	UL function and manual dexterity/ activities and participation	9–10 total minutes (3.5 min test)/seated position	Conventional ARAT and FMA-UE/SUS and Likert questionnaire
Semi-immersive	VR-BBT	Stroke	Cho et al. (2016)/South Korea [26]	*n* = 9 patients/67 ± 8 years (5M/4F)	Observational, cross-sectional design	UL motor function, grasping ability, manual dexterity/activities and participation	32 total minutes estimated (2 min test)/seated position	Conventional BBT
Semi-immersive	VR-BBT	Cervical spinal cord injury, Guillain-Barré syndrome and Sequelae of infectious meningoencephalitis	Álvarez-Rodríguez et al. (2020)/Spain [27]	*n* = 12 patients/22.66 ± 12.99 years (9M/3F)	Cross-sectional; mixed-methods validation.	Gross manual dexterity, presence, usability/activities and participation	3 min estimated/seated position	Conventional BBT
Non-immersive	VR-BBT	Stroke	McKenzie et al. (2017)/United States [28]	*n* = 40 patients/58 ± 14 years (29M/11F)	Cross-sectional study	UL function, accuracy and speed/activities and participation	3 min per hand estimated/seated position	Conventional BBT, ARAT and BI
Non-immersive	VPIT	Stroke	Tobler-Ammann et al. (2016)/Switzerland [29]	*n* = 31 patients/62.7 ± 15.1 years (23M/8F)	Observational study of reliability and validity	Manual dexterity, grasp force, kinematics/body functions and activities and participation	Not estimated time reported/seated position	Conventional NHPT and BBT
Non-immersive	VPIT	Stroke	Kanzler et al. (2020)/ Switzerland [30]	*n* = 23 patients/59 median years (53.5–68.5 years 25th–75th percentile) (9M/14F)	Observational study	Arm-hand sensorimotor control, grasp force/body functions and activities and participation	Not estimated time reported/seated position	Conventional NHPT, BBT, ARAT, FMA-UE, MAS, MoCA and EmNSA
Non-immersive	VPIT	Multiple Sclerosis	Kanzler et al. (2024)/ Switzerland [31]	*n* = 31 patients/56 ± 19.5 years (16M/15F)	Observational study of clinometric/psychometric evaluation	UL sensorimotor and manipulation function and grip force control/body functions and activities and participation	Not estimated time reported/seated position	Conventional BBT, NHPT/SUS questionnaire
**Virtualized Systems Intended for UL Assessment**								
**Type of VR**	**VR Test/Scale**	**Disease**	**Authors (year)/Country**	Sample Size/Mean Age ± SD * (M/F)	**Study Design**	**Domains Assessed Linked to UL/ICF Domain**	**Estimated Administration Time/Patient Position**	**Comparator Measures/Other Scales (if Applied)**
Immersive	VET	Stroke	Lee et al. (2010)/South Korea [32]	*n* = 30 patients/57 ± 10.38 years (23M/7F)	Prospective, cross-sectional, case–control study	Proprioception, visuospatial and sensorimotor integration/body function	Not estimated time reported/seated position	Conventional WMFT, JTHFT and FMA-UE/SSQ questionnaire
Semi-immersive	VOTA	Stroke	Adams et al. (2015)/United States [33]	*n* = 14 patients/69 median years (range 48–87) (4M/10F)	Observational and longitudinal validation study	Gross motor coordination, speed and movement smoothness, Functional movement patterns in ADL/activities and participation	1 h estimated (total session)/seated position	Conventional WMFT
Semi-immersive	SaeboVR^®^(Saebo, Inc., Charlotte, NC, USA)	Stroke	Adams et al. (2019)/United States [34]	*n* = 17 patients/67 median years (range 25–83) (10M/7F)	Cross-sectional study	Movement speed and smoothness, manual dexterity, UL kinematics, functional grasp–release/activities and participation	Not estimated time reported/seated position	Conventional WMFT, FMUE, BBT and MAL
Semi-immersive	Virtual target-to-target pointing task	Stroke	Hussain et al. (2019)/Sweden [35]	*n* = 64 patients/65.7 ± 13.4 years (38M/26F)	Observational, cross-sectional analysis	Movement execution, coordination and control/body functions	Not estimated time reported/seated position	Conventional FMA-UE and ARAT
Semi-immersive	Computerized exergaming system	Parkinson’s disease	Alavian et al. (2024)/Iran [36]	*n* = 33 patients/58.8 ± 7.69 years (19M/14F)	Observational, reliability and validation study	Manual coordination, symmetric and asymmetric movement patterns, kinematic performance/body functions and activities and participation	Not estimated time reported/seated position	Conventional BBT, NHPT, ARAT, RSRD, HAMA and PSMH
Non-immersive	Multi-joint arm exoskeleton	Stroke	Grimm et al. (2021)/ Germany [37]	*n* = 19 patients/56 ± 11 years (11M/8F)	Observational clinical validation study	Joint range of motion and manual grasp/body functions	11 min estimated (6 min test)/seated position	Conventional FMA-UE
Non-immersive	USE-IT^® ^(Upper Extremity Smart Exercises-Innovative Treatment; UDO GAMES Software, Patent No. TR 2015 10835 B, Türkiye)	Stroke	Murat et al. (2025)/ Turkey [38]	*n* = 33 patients/57 median years (IQR 49.50–63.50) (19M/14F)	Observational cross-sectional study	Motor execution and coordination, movement speed/body functions and activities and participation	2 min estimated/seated position	Conventional ARAT, WMFT, FMA-UE, ABILHAND and ACTIVLIM

Notes. * Age is presented as mean ± SD unless otherwise specified. Alternative descriptive measures (e.g., median or range) are indicated where applicable. Colours. Green: virtualized conventional tests/scales for UL assessment; blue: virtualized systems intended for UL assessment; yellow: immersive VR; brown: semi-immersive VR; red: non-immersive VR. ABILHAND: ABILHAND Questionnaire; ACTIVLIM: Activity Limitations Measure; ARAT: Action Research Arm Test; ARAT-VR: Action Research Arm Test Virtual Reality; BBT: Box and Blocks Test; EmNSA: Erasmus Modification of the Nottingham Sensory Assessment; F: female; FMA-UE: Fugl-Meyer Assessment for the Upper Extremity; ICF: International Classification of Functioning; IMI: Intrinsic Motivation Inventory; IQR: Interquartile Range; JTHFT: Jebsen–Taylor Hand Function Test; M: male; MAL: Motor Activity Log; MAS: Modified Ashworth Scale; MMSE: Mini-Mental State Examination; MoCA: Montreal Cognitive Assessment; SD: standard deviation; SSQ: Simulator Sickness Questionnaire; SUS: System Usability Scale; UL: upper limb; USE-IT^®^: Upper Extremity Smart Exercises-Innovative Treatment; VET: Virtual Environment Technique; VOTA: Virtual Occupational Therapy Assistant; VPIT: Virtual Peg Insertion Test; VR: virtual reality; VR-BBT: Virtual Reality Box and Blocks Test; VR-FTT: Virtual Reality Finger Tapping Test; VRUPT: Virtual Reality Upper Extremity Tester; WMFT: Wolf Motor Function Test.

**Table 2:** GRADE recommendation scales.

**Virtualized Conventional Tests/Scales**						
**Type of VR**	**VR Test/Scale**	**Disease**	**Authors (Year)**	**Level of Evidence**	**GRADE Level** **(Recommendation)**	Justification *
Immersive	VR-FTT	Parkinson’s disease	Sanmartín et al. (2013) [19]	Very Low	B Potentially Recommended (Promising but incomplete evidence)	Overall high reliability was reported, but estimates lacked a specific sample size calculation and were not derived solely from the very small Parkinson’s disease subgroup (*n* = 10). Furthermore, construct validity and responsiveness were not assessed, limiting the evaluation of its measurement properties.
Immersive	VR-BBT	Parkinson’s disease	Oña et al. (2020) [20]	Low	B Potentially Recommended (Promising but incomplete evidence)	Moderate to high reliability and correlation values were reported, but the evidence quality was downgraded by risk of bias and imprecision from an incorrect test–retest interval. Furthermore, the lack of a sample size calculation and small sample (*n* = 20) limit the findings’ robustness, while the absence of a priori hypotheses compromises construct validity interpretability.
Immersive	VR-BBT	Stroke	Everard et al. (2022) [21]	Low	B Potentially Recommended (Promising but incomplete evidence)	Strong correlations with the conventional BBT (r = 0.75–0.89) and excellent ICC values (>0.80) were reported. However, the evidence was downgraded due to a small stroke sample (*n* = 22) lacking a sample size calculation, and limited measurement error interpretability restricts determining meaningful functional changes. Although construct validity hypotheses were formulated, they lacked the quantified numerical thresholds recommended by COSMIN.
Immersive	VR-BBT	Stroke	Dong et al. (2023) [22]	Very Low	B Potentially Recommended (Promising but incomplete evidence)	Evidence was downgraded due to potential bias. Despite high results of construct validity, a lack of sample size calculation and a small sample (*n* = 16) limit interpretation regarding meaningful patient improvement. Furthermore, the absence of a priori hypotheses compromises the interpretability of the results.
Immersive	VR-BBT Hand-tracking version	Stroke	Everard et al. (2024) [23]	Low	B Potentially Recommended (Promising but incomplete evidence)	Although sufficient reliability and strong validity were observed, the lack of robust content validity involving patients limits the relevance of the instrument to patient experience. Furthermore, there is a potential risk of bias related to the small sample size derived from the sample size calculation.
	VR-BBT Controller version			Very Low	C Not Recommended (Evidence shows inadequate psychometric performance)	Evidence was downgraded due to severe risk of bias. Despite acceptable reliability, from a patient-centred perspective, controller-based interaction may not accurately reflect natural hand movements, reducing the ability to capture meaningful functional performance. Furthermore, a small sample and the absence of a sample size calculation limit the results’ interpretability.
Immersive	VR-BBT Physical interaction version	Stroke	Yun et al. (2025) [24]	Very Low	B Potentially Recommended (Promising but incomplete evidence)	Evidence was downgraded due to severe risk of bias. While high reliability was reported in stroke patients, a small sample without a sample size calculation limits its robustness. Furthermore, evaluating reliability via repeated trials within a single session restricts the results’ interpretability.
	VR-BBT No Physical interaction version			Very Low	B Potentially Recommended (Promising but incomplete evidence)	
Immersive	ARAT-VR	Stroke	Burton et al. (2022) [25]	Low	B Potentially Recommended (Promising but incomplete evidence)	For the stroke sample (*n* = 30), ARAT-VR showed strong paretic hand associations with conventional ARAT (r = 0.83–0.84) and excellent test–retest reliability (ICC = 0.99). However, evidence was downgraded because the sample size calculation only addressed construct validity. Content validity was rated doubtful, as it relied solely on clinicians’ task-difficulty ratings relative to the original test, lacking patient involvement or qualitative assessment of relevance, comprehensiveness, and comprehensibility.
Semi-immersive	VR-BBT	Stroke	Cho et al. (2016) [26]	Very low	B Potentially Recommended (Promising but incomplete evidence)	The VR-BBT strongly correlated with the conventional BBT for the hemiplegic side (r = 0.788, *p* = 0.012), but evidence was downgraded due to potential bias and severe imprecision from a small stroke sample (*n* = 9) lacking a sample size calculation. Additionally, construct validity interpretability was compromised by the absence of a priori hypotheses.
Semi-immersive	VR-BBT	Cervical spinal cord injury, Guillain-Barré syndrome, Sequelae of infectious meningoencephalitis	Alvarez-Rodríguez et al. (2020) [27]	Very Low	B Potentially Recommended (Promising but incomplete evidence)	In this sample (*n* = 12), the study omitted a separate patient-only correlation for construct validity, preventing confident validation for this population. Additionally, the evidence is limited by severe risk of bias and imprecision due to the very small sample. Furthermore, although hypotheses were formulated, they lacked the quantified numerical thresholds recommended by COSMIN.
Non-immersive	VR-BBT	Stroke	McKenzie et al. (2017) [28]	Low	B Potentially Recommended (Promising but incomplete evidence)	This single cross-sectional study (*n* = 40) showed strong correlations with the FMA and ARAT, but a lack of sample size calculation introduces a potential risk of bias that limits interpretation regarding meaningful functional improvement. Furthermore, although construct validity hypotheses were formulated, they lacked the quantified numerical thresholds recommended by COSMIN.
Non-immersive	VPIT	Stroke	Tobler-Ammann et al. (2016) [29]	Very Low	C Not Recommended (Evidence shows inadequate psychometric performance)	In chronic stroke patients (*n* = 31), the VPIT showed low correlation with the NHPT and BBT, indicating limited validity and inconsistent test–retest reliability. The evidence was downgraded due to severe risk of bias and imprecision stemming from the small sample and lack of a sample size calculation. Furthermore, while construct validity hypotheses were formulated, they lacked the quantified numerical thresholds recommended by COSMIN.
Non-immersive	VPIT	Stroke	Kanzler et al. (2020) [30]	Very Low	C Not Recommended (Evidence shows inadequate psychometric performance)	Only 23 of 30 participants completed the VPIT protocol due to task-related limitations. While some VPIT metrics demonstrated moderate-to-high reliability and construct validity, the evidence was downgraded due to potential risk of bias and severe imprecision from the small sample and lack of a sample size calculation.
Non-immersive	VPIT	Multiple Sclerosis	Kanzler et al. (2024) [31]	Very Low	C Not Recommended (Evidence shows inadequate psychometric performance)	Evidence was downgraded due to the potential risk of bias due to the lack of a sample size calculation. While the VPIT was strongly associated with functional measures, its reliability and measurement error were not studied in this sample. Although construct validity and responsiveness hypotheses were formulated, they lacked the quantified numerical thresholds recommended by COSMIN.
**Virtualized Systems Intended for UL Assessment**						
**Type of VR**	**VR Test/Scale**	**Disease**	**Authors (Year)**	**Level of Evidence**	**GRADE Level** **(Recommendation)**	Justification *
Immersive	VET	Stroke	Lee et al. (2010) [32]	Very Low	B Potentially Recommended (Promising but incomplete evidence)	Although reliability was reported as high, test–retest reliability was assessed using Pearson correlation coefficients, resulting in an inadequate methodological rating according to COSMIN. Furthermore, the authors omitted the statistical methodology, correlation coefficients, and significance values of the comparator outcomes, limiting interpretation and reproducibility. Additionally, the study included a small sample without a sample size calculation.
Semi-immersive	VOTA	Stroke	Adams et al. (2015) [33]	Very low	B Potentially Recommended (Promising but incomplete evidence)	Evidence confidence was reduced by a small sample (*n* = 14), lacking a sample size calculation and doubtful methodological quality. Furthermore, although construct validity hypotheses were formulated, they lacked the quantified numerical thresholds recommended by COSMIN.
Semi-immersive	SaeboVR^®^	Stroke	Adams et al. (2019) [34]	Low	B Potentially Recommended (Promising but incomplete evidence)	The small sample size (*n* = 15) raises concerns regarding precision and potential risk of bias. Furthermore, although hypotheses were formulated for construct validity, these were limited exclusively to the relationship with WMFT-TIME. This lack of comprehensive a priori hypothesis specification introduces methodological limitations and warrants downgrading the evidence.
Semi-immersive	Virtual target-to-target pointing task	Stroke	Hussain et al. (2019) [35]	Very Low	B Potentially Recommended (Promising but incomplete evidence)	Although statistically significant associations with FMA-UE and ARAT were reported, the certainty is reduced by methodological concerns (including small sample, the absence of sample size calculation, and reliance on a single measurement time point. Finally, hypotheses were formulated for construct validity, but they were not quantified with predefined numerical thresholds.
Semi-immersive	Computerized exergaming system	Parkinson’s disease	Alavian et al. (2024) [36]	Very Low	B Potentially Recommended (Promising but incomplete evidence)	The study reports strong reliability results. However, the evidence is limited by the small sample size (*n* = 33), without a sample size calculation, and the absence of construct validity and responsiveness, limiting the evaluation of its measurement properties.
Non-immersive	Multi-joint arm exoskeleton	Stroke	Grimm et al. (2021) [37]	Very Low	B Potentially Recommended (Promising but incomplete evidence)	Kinematic measures obtained from VR showed dispersed correlations with FMA-UE scores (r = 0.54 to 0.79). However, the evidence is limited by the small sample size (*n* = 19) and the scarcity of a sample size calculation. Furthermore, regarding construct validity, no a priori hypotheses were formulated.
Non-immersive	USE-IT^®^	Stroke	Murat et al. (2025) [38]	Very Low	B Potentially Recommended (Promising but incomplete evidence)	The study shows positive construct validity results, with the USE-IT^®^ Global Reaching Map, but the evidence is based on only 33 patients and a scarce sample size calculation. Furthermore, regarding construct validity, no a priori hypotheses were formulated, which further compromises the interpretability of the results.

Notes: * None of the included studies evaluated measurement properties such as content validity, structural validity, internal consistency, or cross-cultural validity, and furthermore, none used the same device or comparable methodologies; this represents a major methodological limitation, introduces a potential risk of bias, and leads to inconsistency, ultimately resulting in low or very low quality of evidence according to GRADE. Colours: Green: virtualized conventional tests/scales for UL assessment; blue: virtualized systems intended for UL assessment; yellow: immersive VR; brown: semi-immersive VR; red: non-immersive VR. ARAT-VR: Virtual Reality Action Research Arm Test; FMA-UE: Fugl-Meyer Assessment for the Upper Extremity; ICC: intraclass correlation coefficient; USE-IT^®^: Upper Extremity Smart Exercises-Innovative Treatment; VET: Virtual Environment Technique; VOTA: Virtual Occupational Therapy Assistant; VPIT: Virtual Peg Insertion Test; VR: virtual reality; VR-BBT: Virtual Reality Box and Blocks Test; VR-FTT: Virtual Reality Finger Tapping Test; WMFT: Wolf Motor Function Test.

**Table 3:** SWOT analysis of UL VR assessment scales in neurorehabilitation.

**Strengths**	**Weaknesses**
• Objective, high-resolution quantitative metrics. • Automatic data recording and immediate feedback. • Ability to capture movement quality and kinematic performance. • Potential for enhanced ecological validity. • Increase patient motivation and adherence through significant tasks.	• Incomplete psychometric validation according to COSMIN recommendations. • Predominantly low to very low certainty of evidence due to methodological limitations and small sample sizes. • High heterogeneity in devices, protocols and outcome metrics.
**Opportunities**	**Threats**
• Methodological standardization and validation across different neurological populations. • Development of patient-centred validation frameworks. • Potential for longitudinal monitoring and home-based assessment. • Development of normative datasets and clinically meaningful change thresholds.	• Premature clinical implementation despite insufficient evidence. • Barriers related to cost, accessibility, and technical requirements. • Potential misalignment with clinical practice standards. • Need for healthcare professional training in technology use and findings interpretation.

## Data Availability

The original contributions presented in this study are included in the article/Supplementary Material. Further inquiries can be directed to the corresponding authors.

## Supplementary Materials

The following supporting information can be downloaded at: https://www.mdpi.com/article/10.3390/neurolint18080142/s1, Supplementary Material Table S1: Guideline for reporting systematic reviews of outcome measurement instruments (PRISMA-COSMIN).

## References

[1] GBD 2021 Nervous System Disorders Collaborators (2024). Global, regional, and national burden of disorders affecting the nervous system, 1990–2021: A systematic analysis for the Global Burden of Disease Study 2021. Lancet Neurol..

[2] Burridge J., Alt Murphy M., Buurke J., Feys P., Keller T., Klamroth-Marganska V., Lamers I., McNicholas L., Prange G., Tarkka I. (2019). A systematic review of international clinical guidelines for rehabilitation of people with neurological conditions: What recommendations are made for upper limb assessment?. Front. Neurol..

[3] World Health Organization (2024). Global Report on Neurological Disorders: Navigating the Future of Neurological Conditions.

[4] Prange-Lasonder G.B., Murphy M.A., Lamers I., Hughes A.M., Buurke J.H., Feys P., Keller T., Klamroth-Marganska V., Tarkka I.M., Timmermans A. (2021). European evidence-based recommendations for clinical assessment of upper limb in neurorehabilitation (CAULIN): Data synthesis from systematic reviews, clinical practice guidelines and expert consensus. J. Neuroeng. Rehabil..

[5] World Health Organization (2001). International Classification of Functioning, Disability and Health: ICF.

[6] Demers M., Levin M.F. (2017). Do activity level outcome measures commonly used in neurological practice assess upper-limb movement quality?. Neurorehabilit. Neural Repair.

[7] Rozaire J., Paquin C., Henry L., Agopyan H., Bard-Pondarré R., Naaim A., Duprey S., Chaleat-Valayer E. (2024). A systematic review of instrumented assessments for upper limb function in cerebral palsy: Current limitations and future directions. J. Neuroeng. Rehabil..

[8] Fernández-González P., Carratalá-Tejada M., Monge-Pereira E., Collado-Vázquez S., Baeza P.S.-H., Cuesta-Gómez A., Oña-Simbaña E.D., Jardón-Huete A., Molina-Rueda F., de Quirós C.B.-B. (2019). Leap motion controlled video game-based therapy for upper limb rehabilitation in patients with Parkinson’s disease: A feasibility study. J. Neuroeng. Rehabil..

[9] Alrashidi M.M., Alanazi A.S., Alkhannani S., Alharbi A., Almuayrifi S., Alhammad A.A., Alhammad S.A., Mashabi A.S. (2025). Virtual reality as an assessment tool in neurorehabilitation: A scoping review of current evidence and future directions. BMC Sports Sci. Med. Rehabil..

[10] Ortiz Gutiérrez R.M., Bermejo Franco A., Cano de la Cuerda R., Cano de la Cuerda R. (2019). Realidad virtual y videojuegos. Nuevas Tecnologías en Neurorrehabilitación. Aplicaciones Diagnósticas y Terapéuticas.

[11] Nieto-Escamez F., Cortés-Pérez I., Obrero-Gaitán E., Fusco A. (2023). Virtual reality applications in neurorehabilitation: Current panorama and challenges. Brain Sci..

[12] Ding K., Ma Y., Zhang L., Gu Y., Pan H., Gu Z.E., Zhang H. (2025). Patient-centered insights into virtual reality rehabilitation for stroke: A systematic review and qualitative meta-synthesis. J. Neuroeng. Rehabil..

[13] Hao J., Li Y., Remis A., He Z., Yao Z., Pu Y. (2024). Performance-based outcome measures of upper extremity in virtual reality and telerehabilitation: A systematic review. Neurol. Sci..

[14] Najsrova A., Banikova S., Szegedi I., Horakova N., Hotkova D., Vitova K., Fiedorova I., Trda J., Volny O. (2026). Patient attitudes toward virtual reality telerehabilitation after subacute stroke: A follow-up study. Bratisl. Med. J..

[15] Khan A., Podlasek A., Somaa F. (2023). Virtual reality in post-stroke neurorehabilitation: A systematic review and meta-analysis. Top. Stroke Rehabil..

[16] Elsman E.B.M., Mokkink L.B., Terwee C.B., Beaton D., Gagnier J.J., Tricco A.C., Baba A., Butcher N.J., Smith M., Hofstetter C. (2024). Guideline for reporting systematic reviews of outcome measurement instruments (OMIs): PRISMA-COSMIN for OMIs 2024. Qual. Life Res..

[17] Mokkink L.B., Elsman E., Terwee C.B. (2024). COSMIN guideline for systematic reviews of patient-reported outcome measures version 2.0. Qual. Life Res..

[18] GRADE Working Group (2004). Grading quality of evidence and strength of recommendations. BMJ.

[19] Sanmartín G., Flores J., Robles-García V., Arias P., Cudeiro J. (2013). Low-cost virtual reality system in evaluation of rhythmic motor patterns in elderly and Parkinson’s disease patients. Presence.

[20] Oña E.D., Jardón A., Cuesta-Gómez A., Sánchez-Herrera-Baeza P., Cano-de-la-Cuerda R., Balaguer C. (2020). Validity of a fully-immersive VR-based version of the Box and Blocks Test for upper limb function assessment in Parkinson’s disease. Sensors.

[21] Everard G., Otmane-Tolba Y., Rosselli Z., Pellissier T., Ajana K., Dehem S., Auvinet E., Edwards M.G., Lebleu J., Lejeune T. (2022). Concurrent validity of an immersive virtual reality version of the Box and Block Test to assess manual dexterity among patients with stroke. J. Neuroeng. Rehabil..

[22] Dong Y., Liu X., Tang M., Huo H., Chen D., Wu Z., An R., Fan Y. (2023). A haptic-feedback virtual reality system to improve the Box and Block Test (BBT) for upper extremity motor function assessment. Virtual Real..

[23] Everard G., Burton Q., Van de Sype V., Bibentyo T.N., Auvinet E., Edwards M.G., Batcho C.S., Lejeune T. (2024). Extended reality to assess post-stroke manual dexterity: Contrasts between the classic Box and Block Test, immersive virtual reality with controllers, hand-tracking, and mixed-reality tests. J. Neuroeng. Rehabil..

[24] Yun S.J., Kim J.H., Lee J.H., Kim D.E., Oh B.-M., Lee W.H., Kwon B.S., Gil Seo H. (2025). Reliability and validity of virtual reality Box and Block Test in healthy adults and patients with stroke: A prospective multicenter exploratory cross-sectional study. J. Neuroeng. Rehabil..

[25] Burton Q., Lejeune T., Dehem S., Lebrun N., Ajana K., Edwards M.G., Everard G. (2022). Performing a shortened version of the Action Research Arm Test in immersive virtual reality to assess post-stroke upper limb activity. J. Neuroeng. Rehabil..

[26] Cho S., Kim W.S., Paik N.J., Bang H. (2016). Upper-limb function assessment using VBBTs for stroke patients. IEEE Comput. Graph. Appl..

[27] Alvarez-Rodríguez M., López-Dolado E., Salas-Monedero M., Lozano-Berrio V., Ceruelo-Abajo S., Gil-Agudo A., de Los Reyes-Guzmán A. (2020). Concurrent validity of a virtual version of Box and Block Test for patients with neurological disorders. World J. Neurosci..

[28] McKenzie A., Dodakian L., See J., Le V., Quinlan E.B., Bridgford C., Head D., Han V.L., Cramer S.C. (2017). Validity of robot-based assessments of upper extremity function. Arch. Phys. Med. Rehabil..

[29] Tobler-Ammann B.C., De Bruin E.D., Fluet M.C., Lambercy O., De Bie R.A., Knols R.H. (2016). Concurrent validity and test-retest reliability of the Virtual Peg Insertion Test to quantify upper limb function in patients with chronic stroke. J. Neuroeng. Rehabil..

[30] Kanzler C.M., Schwarz A., Held J.P.O., Luft A.R., Gassert R., Lambercy O. (2020). Technology-aided assessment of functionally relevant sensorimotor impairments in arm and hand of post-stroke individuals. J. Neuroeng. Rehabil..

[31] Kanzler C.M., Armand T., Simovic L., Sylvester R., Domnik N., Eilfort A.M., Rohner C., Gassert R., Gonzenbach R., Lambercy O. (2024). Influence of virtual reality and task complexity on digital health metrics assessing upper limb function. J. Neuroeng. Rehabil..

[32] Lee K.H., Ku J., Jo S.W., Kim S.I., Song J.Y., Park Y.J., Kim H.J., Kang Y.J. (2010). Upper extremity proprioceptive assessment test using virtual environment technique in patients with stroke. J. Korean Acad. Rehabil. Med..

[33] Adams R.J., Lichter M.D., Krepkovich E.T., Ellington A., White M., Diamond P.T. (2015). Assessing upper extremity motor function in practice of virtual activities of daily living. IEEE Trans. Neural Syst. Rehabil. Eng..

[34] Adams R.J., Ellington A.L., Armstead K., Sheffield K., Patrie J.T., Diamond P.T. (2019). Upper extremity function assessment using a glove orthosis and virtual reality system. OTJR.

[35] Hussain N., Sunnerhagen K.S., Alt Murphy M. (2019). End-point kinematics using virtual reality explaining upper limb impairment and activity capacity in stroke. J. Neuroeng. Rehabil..

[36] Alavian S., Taghizade G., Mahdizade H., Behzadipour S. (2024). A computerized assessment tool for upper extremity motor performance in individuals with Parkinson’s disease. Biomed. Signal Process Control.

[37] Grimm F., Kraugmann J., Naros G., Gharabaghi A. (2021). Clinical validation of kinematic assessments of post-stroke upper limb movements with a multi-joint arm exoskeleton. J. Neuroeng. Rehabil..

[38] Murat G., Doğan M., Onursal Kılınç Ö., Aksu Yıldırım S., Kılınç M. (2025). Upper extremity function assessment test using virtual environment technique in stroke survivors. Somatosens. Mot. Res..

[39] Fugl-Meyer A.R., Jääskö L., Leyman I., Olsson S., Steglind S. (1975). The post-stroke hemiplegic patient. 1. A method for evaluation of physical performance. Scand. J. Rehabil. Med..

[40] Mathiowetz V., Volland G., Kashman N., Weber K. (1985). Adult norms for the Box and Block Test of manual dexterity. Am. J. Occup. Ther..

[41] Folstein M.F., Folstein S.E., McHugh P.R. (1975). Mini-mental state. A practical method for grading the cognitive state of patients for the clinician. J. Psychiatr. Res..

[42] Nasreddine Z.S., Phillips N.A., Bédirian V., Charbonneau S., Whitehead V., Collin I., Cummings J.L., Chertkow H. (2005). The Montreal Cognitive Assessment, MoCA: A brief screening tool for mild cognitive impairment. J. Am. Geriatr. Soc..

[43] Lyle R.C. (1981). A performance test for assessment of upper limb function in physical rehabilitation treatment and research. Int. J. Rehabil. Res..

[44] Wolf S.L., Catlin P.A., Ellis M., Archer A.L., Morgan B., Piacentino A. (2001). Assessing Wolf motor function test as outcome measure for research in patients after stroke. Stroke.

[45] Brunnstrom S. (1970). Movement Therapy in Hemiplegia: A Neurophysiological Approach.

[46] Brott T., Adams H.P., Olinger C.P., Marler J.R., Barsan W.G., Biller J., Spilker J., Holleran R., Eberle R., Hertzberg V. (1989). Measurements of acute cerebral infarction: A clinical examination scale. Stroke.

[47] Hoehn M.M., Yahr M.D. (1967). Parkinsonism: Onset, progression and mortality. Neurology.

[48] Goetz C.G., Tilley B.C., Shaftman S.R., Stebbins G.T., Fahn S., Martinez-Martin P., Poewe W., Sampaio C., Stern M.B., Dodel R. (2008). Movement Disorder Society-sponsored revision of the Unified Parkinson’s Disease Rating Scale (MDS-UPDRS): Scale presentation and clinimetric testing results. Mov. Disord..

[49] Kurtzke J.F. (1983). Rating neurologic impairment in multiple sclerosis: An Expanded Disability Status Scale (EDSS). Neurology.

[50] Kirshblum S.C., Burns S.P., Biering-Sørensen F., Donovan W., Graves D.E., Jha A., Johansen M., Jones L., Krassioukov A., Mulcahey M.J. (2011). International standards for neurological classification of spinal cord injury (revised 2011). J. Spinal Cord. Med..

[51] Demeurisse G., Demol O., Robaye E. (1980). Motor evaluation in vascular hemiplegia. Eur. Neurol..

[52] Morrison M.W., Gregory R.J., Paul J.J. (1979). Reliability of the Finger Tapping Test and a note on sex differences. Percept. Mot. Ski..

[53] Mathiowetz V., Weber K., Kashman N., Volland G. (1985). Adult norms for the Nine Hole Peg Test of finger dexterity. Occup. Ther. J. Res..

[54] Rose-Dulcina K., Armand S., Cacioppo M. (2025). Using game performance measures to assess upper limb motor function in children with neuromotor disorders: A systematic review. J. Neuroeng. Rehabil..

[55] Hao J., Crum G., Siu K.C. (2024). Effects of virtual reality on stroke rehabilitation: An umbrella review of systematic reviews. Health Sci. Rep..

[56] Huang J., Wei Y., Zhou P., He X., Li H., Wei X. (2025). Effect of home-based virtual reality training on upper extremity recovery in patients with stroke: Systematic review. J. Med. Internet Res..

[57] Herrera-Rojas A., Moreno-Molina A., García-García E., Molina-Rodríguez N., Cano-de-la-Cuerda R. (2025). Effects of telerehabilitation platforms on quality of life in people with multiple sclerosis: A systematic review of randomized clinical trials. NeuroSci.

[58] Cano-De-La-Cuerda R., Blázquez-Fernández A., Marcos-Antón S., Sánchez-Herrera-Baeza P., Fernández-González P., Collado-Vázquez S., Jiménez-Antona C., Laguarta-Val S. (2024). Economic cost of rehabilitation with robotic and virtual reality systems in people with neurological disorders: A systematic review. J. Clin. Med..

[59] Tannus J., Naves E.M., Morere Y. (2024). Post-stroke functional assessments based on rehabilitation games and their correlation with clinical scales: A scoping review. Med. Biol. Eng. Comput..

[60] Röseler L., Kaiser L., Doetsch C., Klett N., Seida C., Schütz A., Aczel B., Adelina N., Agostini V., Alarie S. (2024). The Replication Database: Documenting the replicability of psychological science. J. Open Psychol. Data.

[61] Maden Ç., Gözaçan Karabulut D., Bağcı B. (2025). Validity and reliability of an immersive virtual reality adaptation of the 6-minute pegboard and ring test. Hand Surg. Rehabil..

[62] Chen Y.C., Liang H.W. (2025). Application of a virtual reality-based measurement of simple reaction time in adults: A psychometric evaluation. Virtual Real..

[63] Taitano R.I., Yough M.G., Hanna K., Korol A.S., Gritsenko V. (2024). Setup for the quantitative assessment of motion and muscle activity during a virtual modified box and block test. J. Vis. Exp..

[64] Wilf M., Korakin A., Bahat Y., Koren O., Galor N., Dagan O., Wright W.G., Friedman J., Plotnik M. (2024). Using virtual reality-based neurocognitive testing and eye tracking to study naturalistic cognitive-motor performance. Neuropsychologia.

[65] FFernández-Cañas M., Ortiz-Gutiérrez R.M., Martín-Casas P., Estrada-Barranco C., Marcos-Antón S., Blázquez-Fernández A., Laguarta-Val S., Jiménez-Antona C., Cano-De-La-Cuerda R. (2025). Cybersickness evaluation in immersive virtual environments: A systematic review with implications for neurological rehabilitation. J. Clin. Med..

[66] Cano-de-la-Cuerda R., Muñoz-Hellín E., Alguacil-Diego I.M., Molina-Rueda F. (2010). Telerrehabilitación y neurología. Rev. Neurol..

[67] Marcos-Antón S., Cano-de-la-Cuerda R., Aranda-Reneo I., Jardón-Huete A., Oña-Simbaña E.D., Oliva-Moreno J. (2026). Cost-effectiveness analysis of the MYO Armband^®^ device in combination with specifically designed video games for upper limb rehabilitation in people with multiple sclerosis. J. Neuroeng. Rehabil..

